# The safety and efficacy of AphtoFix® mouth ulcer cream in the management of recurrent aphthous stomatitis

**DOI:** 10.1186/s12903-016-0177-0

**Published:** 2016-02-11

**Authors:** Amina Sakly, Bart De Wever, Balraj Jutla, Milan Satia, Jean Pierre Bogaert

**Affiliations:** MSI (Medical Sanitizing Innovations) Laboratories AG, Vaduz, Liechtenstein; Bonyf AG, Heiligkreuz 16, FL-9490 Vaduz, Liechtenstein; Ethicare Clinical Trial Services; Satellite, Ahmedabad, 380015 Gujarat India

**Keywords:** Aphthous stomatitis, Aphthous ulcer, Canker sores, Healing

## Abstract

**Background:**

Recurrent Aphthous stomatitis (RAS) is a prevalent ulcerative and painful disorder of the oral cavity with unknown etiology and for which no efficient treatment is currently available.

The present study aimed to evaluate the safety and the efficacy of AphtoFix®, a new mouth ulcer cream that was developed to help treat RAS. Prior to launching the product on the market, two initial safety assessment studies were performed.

**Subjects and methods:**

In a first study, the in vitro biocompatibility of AphtoFix® was evaluated on reconstructed human gingival tissue models according to ISO guidelines 10993. In a second study, the tolerability of AphtoFix® was evaluated in 20 subjects during a 4-weeks daily application in the mouth. The third study investigated both the safety and efficacy of AphtoFix® treatment on 19 patients suffering from RAS. This study was done in compliance with the Helsinki Declaration.

**Results:**

The results of in vitro biocompatibility study showed that AphtoFix® mouth ulcer cream did not induce any detectable cytotoxicity and irritation. These observations were confirmed in the 4 weeks tolerability study where no undesired of adverse reactions were noticed. The results of the post-market clinical efficacy study demonstrated a clear reduction in ulcer size from baseline after 3 days treatment (*p* < 0.05). Pain intensity reduction was also observed in all subjects.

**Conclusion:**

The application of AphtoFix® did not induce any undesired skin or mucosa reactions. These initial findings demonstrate that AphtoFix® is safe and efficient in reducing ulcer size and decreasing the pain intensity induced by ulcers.

**Trial registration:**

Clinical trial Registry India Nr. CTRI201408004918, Date of registration: 22/08/2014

**Electronic supplementary material:**

The online version of this article (doi:10.1186/s12903-016-0177-0) contains supplementary material, which is available to authorized users.

## Introduction

Recurrent aphthous stomatitis (RAS) is a widespread ulcerative disease of the oral mucosa characterized by a painful round, shallow ulcers with well-defined erythematous margin and yellowish-gray pseudomembranous center [1]. RAS affects 5–25 % of the population and more frequent in patients between 10–40 years of age [2–9]. This type of ulcer affects women and individuals of higher socioeconomic levels [2, 10, 11].

RAS is classified as minor, major, and herpetiform. Seventy to 87 % of all RAS cases are minor [12, 13]. After 24 to 48 h preceding the development of a minor aphthous ulcer, subjects may experience a pricking or burning sensation in the mucosa. Typically, the ulcers are less than 1 cm in diameter and less than five occur at any one time. These ulcers are self-limiting and resolve within 7–10 days without scarring [1, 12, 14–16].

Major aphthae are larger and cause deeper ulcers. They usually take more than a month to heal, and frequently leave a scar. These typically develop after puberty with frequent recurrences. Herpetiform ulcerations are the most severe form. It is characterized by small, numerous, 1 to 3 mm lesions that form clusters. They typically heal in less than a month without scarring [17].

RAS is a difficult disorder to treat. All therapies are aimed to decrease the painful symptoms and duration of the ulcers [18, 19]. Pain is subjective and depends on several factors such as personal experience, age, social, ethnic factors, and perceptual abilities [20], and patients may seek advice from a variety of sources as regards appropriate therapy and often self- medicate with a range of agents [21]. Actually, topical or systemic antibacterial such us chlorhexidine, anti-inflammatory, immunomodulatory, or symptomatic treatments are used however such treatments are not totally reliable [12, 19, 22–26].

The inflammatory process plays an important role in RAS. Neutrophils and mononuclear cell infiltration in the lamina propria determine the primary histologic characteristics [1, 27]. Local immune dysfunction exerts also a significant role in the development of RAS [28, 29]. The presence of abnormal oral flora was associated with an aberrant immune and inflammation response that is why oral bacteria play a primary pathogenic role in the development of RAS. Due to the potential multifactorial etiology and pathophysiology, the optimal therapeutic agent should ideally combine several bioactive agents [28, 30].

Several topical agents are available for symptomatic relief such as antibiotics, local anaesthetics, antihistamines, non-steroidal anti-inflammatory drugs, enzymatic preparations, gammaglobulins and immunosuppressants. However, the problem lies that the efficacy of the majority of these agents has not been adequately evaluated in designed and controlled clinical trials [12, 15, 29, 31–34].

AphtoFix® mouth ulcer cream is a new generation of mouth ulcer cream with protective as well as adhesive properties that enhance the healing of the ulcer. The adhesive properties of AphtoFix® are derived from the Cellulose Gum and Calcium/Sodium PVM/MA Copolymer, which modify the cream’s physical properties on contact with moisture to create a thin, elastic cushion against pressure sores. AphtoFix® mouth ulcer cream contains ingredients that activate on contact with the mouths saliva. This enables the cream to create a protective and lasting layer over the ulcer area.

The aim of this study is to evaluate the safety and efficacy of AphtoFix in the management of RAS. The safety of AphtoFix® was assessed firstly by testing the cytotoxicity and irritation on in vitro reconstructed human gingival tissue according to ISO10993 and secondly by applying this product on 20 healthy subjects during 4 weeks.

The clinical efficacy of AphtoFix® was studied on 19 subjects suffering from RAS. The majority of subjects have recurrent aphthous ulceration which tend to be otherwise healthy without signs of systemic disease (minor ulcer). Ulcer parameters such as size, pain intensity and duration of each ulcer are evaluated in this study.

## Methods

### In vitro biocompatibility evaluation according to ISO10993-part1

#### Materials

Used Materials: Reconstructed human gingival epithelial tissues

In this study we used the three dimensional reconstructed human gingival epithelium model (Sterlab, France, batch number 1301 GEN 01) that consists of normal human gingival keratinocytes that were cultured for 5 days on 0.5 cm^2^ polycarbonate filter inserts at the air-liquid interface. The Sterlab gingival model presents a histological morphology comparable to the in vivo human tissue. After 5 days of tissue reconstruction, the gingival tissues were transferred on an agarose gel and incubated at room temperature during 24 h (simulation of shipping conditions). Inserts were removed from the agarose gel and transferred on 1 mL growth medium. Then tissues were placed at 36.5 °C / 5 % CO_2_ for 24 h.

### Method of analysis

- Cytotoxicity assay (according to ISO10993-05 for gingival epithelium)
  Each test substance (AphtoFix®, negative and positive controls) was topically applied on two reconstructed human gingival tissues for 24 h at 36.5 °C / 5 % CO_2_. After exposure, all tissues were rinsed with Phosphate Buffer Saline. Then, cell viability was determined by incubating the tissues for three hours with 300 μL MTT solution (0.5 mg/mL). After MTT incubation, tissues were rinsed 3 times with PBS and consequently extracted with 1 ml of Isopropanol. Optical density was quantified by spectrophotometry at 550 nm wavelength. Quantitative viability was determined as a percentage of negative control.
- Irritation assay (according to ISO10993-10 for gingival epithelium)
  Each test substance (AphtoFix®, negative and positive controls) were topically applied on two reconstructed gingival tissues for 2 h followed by a 42 h recovery incubation at 36.5 °C / 5 % CO2. After exposure, tissues were rinsed with Phosphate Buffer Saline. Then, cell viability was determined by incubating the tissues for three hours with 300 μL MTT solution (0.5 mg/mL). Optical density was quantified by spectrophotometry at 550 nm wavelength. Quantitative viability was determined as a percentage of negative control.

### In vivo safety evaluation

An in vivo clinical trial was conducted on 20 healthy subjects to evaluate the safety of AphtoFix® application during 4 weeks. The study population included 6 males and 14 females. The mean age of this group is 38.45 years. The aim of this study was to examine the tolerability of AphtoFix® mouth ulcer cream according to clinical-dermatological test criteria. Before the commencement of this trial, all participants were examined for eventual skin irritation by 2 specialists in dermatology and Venerology from Dermatest® GmbH Research Institute. Only subjects without any pathological skin disorders were included in the test group. The participants were able to consult the physicians in charge of the trail any day in case of any objective or subjective skin changes were noted.

The test participants were instructed to use the product in the mouth (topical application on the oral mucosa) 2 to 4 times per day during 4 weeks. A small amount of cream was applied on lips, cheek, tongue and jaw. In addition, the participants were also advised to not to use any other skin care product in the test area during the course of the 4 weeks test period.

### In vivo efficacy and safety evaluation of AphtoFix®

#### Study population

The study was performed at the Poojan Multispeciality Hospital and the APL Institute of clinical Laboratory & Research (Ahmedabad-380052, Gujarat, India). 22 subjects were screened and enrolled into the study according to inclusion and exclusion criteria.

Inclusion criteria:

(1) Male or female subjects with 18 – 65 years of age; (2) mouth ulcer size >2 mm at easily accessible location in the mouth allowing easy evaluation and treatment; (3) Subject with start of mouth ulceration within 48 h; (4) Subject who agreed not to use any other medication to treat mouth ulcer during study period; (5) Subject willing to comply with the study schedule and procedures; (6) Subject or legally acceptable representative (LAR) of subject willing to sign and date written informed consent to participate in the study. However, if the subject/LAR of subject would be illiterate, the impartial witness would sign the Informed Consent Form (ICF).

Exclusion criteria:

(1) Subjects with a history of allergy for ingredients present in AphtoFix®®; (2) Subjects with any invasive dental procedures within 2 weeks prior to screening visit; (3) History of uncontrolled chronic disease (e.g. chronic liver disease, chronic renal disease); (4) Use of any other medication to treat mouth ulcer in current episode; (5) Participation in any other clinical study during last 30 days; (6) Subject with any condition which in the opinion of the investigator makes the subject unsuitable for inclusion; (7) Subject is a female who is pregnant or willing to get pregnant, not ready to use contraceptive measures during the trial period, or is breast feeding.

Out of 22 enrolled subjects, 19 subjects were analyzed and 3 subjects were excluded from the analysis since they were lost during follow up and hence not considered for statistical analysis.

#### Ethical approval and informed consent

The present research conformed to the Helsinki Declaration, and ethical clearance and approval letter was obtained from Sangini Hospital Ethics Committee in India issued by the Department of Health and Human Service (DHHS-IORG0007258), Office of Human Research Protection (OHRP-IRB00008709) and Drug Controller General of India (DCGI-ECR/147/Inst/Gj/2013).

All participants received a full explanation of the study and provided written informed consent. Information provided to the subjects was pre-approved by IEC (Indian Ethical committee).

The subjects were informed about the purpose, procedures to be carried out, potential hazards and the subject's right to claim compensation in case of trial related injury and death before participating in the study. The written informed consent form and patient information sheet included all the information required to fulfil the ICH-GCP guidelines and recommendations published by Government of India.

Subjects were allowed to ask questions and clarify their doubts regarding any aspect of the study. Signing the informed consent form meant that the subjects confirmed his voluntary participation and his intention to comply with the protocol and the investigator’s instructions and to answer all questions under the study.

For any change in informed consent form (either due to generation of new information or due to procedural amendments) a prior permission from the IEC and approval from all investigators were considered mandatory.

#### Study protocol

After signing the informed consent document, subjects were screened for demographic data, medical history, clinical and physical examination including vital signs, objective and subjective assessments. Total of 19 subjects were enrolled in the study. As all the subjects had ulcers within 48 h duration prior to screening visit, all the subjects were enrolled on the screening visit, hence screening visit (visit 1) and enrollment visit for all the subjects were same.

Treatment with AphtoFix® mouth ulcer cream was initiated immediately after enrolment. A small amount of cream was collected on the tip of the finger by squeezing and applied on affected area (mouth ulcers) using a swiping action to ensure that it covers the whole area of ulceration. The cream was applied multiple times during the day (up to 4 applications per day) for maximum of 14 days, usually after meals and before going to sleep.

Application of cream was continued by the subjects until reduction in ulcer size and symptoms. During the enrollment visit (visit 2 day1), at each follow-up visit (Visit 3 (day 4) and visit 4 (day 7)) and at the end of study visit (visit 5), a clinical examination including vital signs measurement as well as all subjective and objective assessments were carried out. Subjects were evaluated for physical examination at the time of complete healing of ulcer. Adverse event evaluation was carried out at Visit 3, Visit 4 and Visit 5 (day 14).

Efficacy assessment was done by evaluating the primary outcomes such as the mean of ulcer size reduction, the change in number of ulcers and pain intensity reduction (VAS) assessed at Visit 3 (Day 4), Visit 4 (Day 7), and Visit 5 (Day 14) and compared to baseline. The secondary outcomes are the evaluation of ulcer duration (days needed for ulcer healing), the number and frequency of application obtained from the subject diary and finally the quality of life (QOL). The assessment of QOL was evaluated by using a questionnaire subdivided into 3 domains such as pain and functional level and finally medication and treatment limitation of subjects of during treatment period (Additional file 1: Annex I).

#### Statistical analysis

All measurement variables were expressed as mean and standard deviation whereas categorical variables were expressed as percentage. Efficacy parameters were analyzed using the Wilcoxon matched-pairs signed rank test.

Reduction in pain and in number of ulcer from baseline to each follow up visits (visit 3 and 4) and end of study visit (visit 5) was analyzed using paired “t” test or Wilcoxon test depending upon the distribution of data. Normality test (Kolmogorov-Smirnov test or Shapiro-Wilks test) was used to check the distribution of data.

*P* values less than 0.05 were considered as statistically significant difference. The data were presented as mean ± Standard Deviation/Standard error mean with 95 % confidence interval.

## Results

### In vitro biocompatibility safety assessment

No cytotoxic effect was observed on reconstructed gingival tissues after 24 h of contact with AphtoFix®. The viability was 95.81 % compared to the positive control showing a viability equal to 1.85 %. In addition, the product AphtoFix® was also not considered irritant. Viability of the reconstructed human gingival tissues after 2 h contact with AphtoFix®, followed by a 42 h recovery incubation was 92.55 % compared to the positive control demonstrating a viability equal to 51.75 %. Results are shown on Table 1.

**Table 1:** Cytotoxicity and irritation assays

Assays	Negative control PBS	Positive control SDS 1%	AphtoFix
Cytotoxicity (Viability)	100 %	1.85 %	95.81 %
Irritation (Viability)	100 %	51.75 %	92.55 %

#### In vivo safety assessment of AphtoFix® on healthy subjects

All of the 20 healthy subjects tolerated the product AphtoFix® mouth ulcer cream very well during the course of the four-week test under dermatological and clinical observation. There were no undesired or even pathological reactions. It can therefore be concluded that the use of AphtoFix® mouth ulcer cream does not lead to any undesired skin or mucosa reactions.

#### In vivo clinical efficacy and safety assessment of AphtoFix® on patients suffering from RAS

##### Primary end points

- Demographic characteristics of the study population
  Nineteen patients were enrolled in the study (16 males; 03 females). The mean (± SD) age of study subjects was 27.95 ± 7.75 years (range 18–46).
- Ulcer size reduction
  Out of 19 subjects, 17 subjects were diagnosed with a single ulcer and the remaining 2 subjects had 2 ulcers. All the subjects had ulcer sizes ranging from 4 mm to 7 mm. The gradual reduction in ulcer size was measured after treatment at each post-treatment visit. Significant gradual reduction in ulcer size was observed at each post-treatment visit compared with baseline. At visit 4, all mouth ulcers were completely disappeared in 18 subjects. In the remaining 1 subject, the ulcer disappeared at 13th day. The difference of ulcer size between baseline and visit 4 was statistically different: 4.33 ± 0.91 mm versus 0.25 ± 0.71 mm, *p* < 0.05).
  Since no other medication that could impact mouth ulcer evolution was allowed during the study, the observed efficacy is solely related only to AphtoFix® mouth ulcer cream application. Mean change in ulcer size is showed in Table 2.

**Table 2:** Ulcer size reduction

Study visits	Day 1	Day 4	Day 7
Number of subjects (N)	19	19	19
Mean ulcer size	4.33 ± 0.91	1.19 ± 1.69*	0.25 ± 0.71*
Percentage improvement in ulcer size	0.00 %	72.53 %	94.23 %

Values are expressed as Mean ± SD. *p<0.05 as compared to baseline. Data were analyzed by Wilcoxon matched-pairs signed rank test
- Number of ulcers healed
  A total 21 of mouth ulcers (2 subjects were having 2 ulcer sites) were observed in 19 subjects. Subjects were also instructed to note if the ulcer had healed completely before scheduled visit in subject diary, and to report this immediately to the hospital site before the scheduled visit. Out of 21 mouth ulcers, 38.10 % (*n* = 8) ulcers were healed after 3 days of treatment, 23.81 % (*n* = 5) ulcers were healed after 4 days of treatment, 14.29 % (*n* = 3) ulcers were healed after 5 days of treatment, 19.05 % (*n* = 4) ulcers were healed after 6 days and 4.76 % (*n* = 1) ulcers were healed after 13 days of treatment (Table 3).

**Table 3:** Number of healed ulcer and pain intensity (VAS)

Treatment days	Day 3	Day 4	Day 7	Day 13
Number of healed ulcer (Improvement percentage)	8 (38.10 %)	5 (23.81 %)	4 (19.05 %)	1 (4.76 %)
Mean Pain intensity in (VAS)	6.58 ± 0.90	1.58 ± 2.24*	0.29 ± 0.76*	00

Values are expressed as Mean ± SD. * p<0.05 as compared to baseline. Data were analyzed by Wilcoxon matched-pairs signed rank test
- Pain intensity Assessment by VAS (Visual Analogue Scale)
  A significant reduction in pain intensity score (VAS) was observed compared to baseline (1.58 ± 2.24 and 0.29 ± 0.76 at visit 3 and visit 4 respectively versus 6.58 ± 0.90 at baseline). Percentage of pain intensity improvement were observed at visit 3, visit 4 and visit 5 as 76.00 %, 95.66 % and 100 %, respectively. Percentage improvement in pain assessment is illustrated in Table 3.

##### Secondary endpoints: Quality of Life (QOL)

- Pain and functional limitation assessment
  Both information on pain intensity and functional limitation level assessment were the first domain of QOL questionnaire. Both gradual reduction in pain as well as the level of functional limitation during daily activity was reported at each visit. There was significant reduction in pain and functional level during daily activity at visit 3 (3.16 ± 4.41) and visit 4 (0.71 ± 1.89) as compared to baseline (14.95 ± 3.27). The improvement in pain and functional activity was significantly greater at visit: in fact, pain and functional level were zero at visit 4 in 18 subjects which coincides with complete ulcer healing. Percentage improvement in pain and functional level at visit 3, visit 4 and visit 5 were observed as 78.88 %, 95.22 % and 100 %, respectively.
- Medication and treatment level assessment
  Medication and treatment level was the second domain of QOL questionnaire. Since the scoring for this domain could only be obtained after study treatment, therefore scoring was started from visit 3. The Quality of life (QOL) related to medical treatment was statistically significant at visit 4 (0.29 ± 0.76) as compared to visit 3 (1.58 ± 2.19), indicating early relief from mouth ulcer. The medical treatment requirement was gradually decreased from follow up visit 3 to study visit 4. Percentage of improvement was observed at visit 4 (81.92 %) and visit 5 (100 %).

## Discussion

Although several factors are suspected including genetics, stress, nutritional deficiencies, diet, hormonal changes, and immunological disorders, the cause of RAS is unknown [27, 35, 36]. Due to the indeterminate etiology of RAS, it is difficult to find a definitive cure and current treatments aimed to ameliorate the symptoms. Treatment for RAS is symptomatic; the goals are essentially pain reduction, healing time, number and size of the ulcer, and to increase disease-free periods [37].

Based on the results of several case studies where individual patients reported RAS reduction and concomitant pain relief, we decided to investigate the efficacy of AphtoFix® as a possible candidate treatment because of its known adhesive properties.

AphtoFix® was formulated using a variety of similar ingredients that can be found in denture adhesive creams including PVP. One of the most important applications of PVP, either used alone or in combination with other polymers, is the coating or the encapsulation of tissues, organs or implants. This is because PVP provides a film that is permeable to water and solutes and, at the same time, protects tissue or implant surfaces from insults or other damages like those due to bacterial colonization. Once in contact with the oral mucosa, AphtoFix® creates a thin, elastic cushion on mucosa surface. In addition, the cream acts as a protection against pressure sores and irritation of the nearby gums.

In this study, the application of AphtoFix® mouth ulcer cream demonstrated a clear efficacy in the treatment of RAS, based on objective parameters including of size of ulcer, number of healed ulcer, duration of ulcer, and pain intensity. These findings showed almost a significant reduction of both pain intensity and ulcer size after 4 days of treatment compared to baseline values. Normally, these ulcers are self-limiting and resolve within 7–10 days [1, 12, 14–16].

Several studies investigated the effects of some treatments on pain and healing times. The results showed a significant (*p* < 0.05) reduction in pain and healing time when Aphtheal was applied to the ulcer 4 times a day from ulcer onset until healing [38–40]. The study of Fontes et al. showed that colchicine improved the symptoms of RAS (pain and size) in 63 % of cases [41]. Symptoms of aphthous ulcer were improved after the application of Pentoxifylline and the response rates varied between 36 % and 63 % this were rapidly followed by recurrences as soon as the medication was stopped [42, 43]. Steroids are usually used for a short period (up to 1 month). Efficacy rate is reported between 30 % and 50 % [34]. Porter et al. showed that HybenX application reduced the painful symptoms of RAS [44]. Moghadamnia et al. evaluated the efficacy of licorice bioadhesive hydrogel patches for controlling the pain and reducing the healing time of RAS. The study showed that licorice bioadhesive can be effective in the reduction of the pain and the sizes of the inflammatory halo [45]. In another clinical trial using herbal options muco-bioadhesive containing ginger officinale extract was indicated relieving pain of RAS but had no efficacy on ulcer diameter, inflamed halo and healing time when compared to a placebo [46].

RAS causes not only pain but could also decrease the Quality of Life (QOL) by interfering with swallowing, drinking, eating and even speaking [47, 48]. A standardized questionnaire was included in our study. The analysis showed a significant improvement of QOL.

## Conclusion

The present study findings demonstrated that the topical application of AphtoFix® mouth ulcer cream could effectively reduce the ulcer size and alleviate pain. As no adverse or serious adverse events were reported during the entire study period of both human clinical trials, AphtoFix® can be considered as a safe and efficient product for the treatment of minor RAS.

## Additional file

Additional file 1:
**Quality of Life (QOL).** (DOCX 13 kb)

## Abbreviations

RAS: recurrent aphthous stomatitis
VAS: visual analog scale
QOL: Quality of life
PVP: polyvinylpyrrolidone
PVM/MA: methyl vinyl ether/ maleic anhydride
