# Supplementary material for: The safety and efficacy of AphtoFix® mouth ulcer cream in the management of recurrent aphthous stomatitis
Source: BMC Oral Health. 2016 Feb 11;16:17. doi: 10.1186/s12903-016-0177-0 (PMC4750241; doi:10.1186/s12903-016-0177-0)
Supplement: Additional file 1: — Quality of Life (QOL). (DOCX 13 kb) [file 12903_2016_177_MOESM1_ESM.docx]

**Annex I Quality of Life (QOL) Questionnaire**

| **QUESTIONS** | | **RATINGS** | | | | |
| --- | --- | --- | --- | --- | --- | --- |
| **Pain and Functional Limitation:** | | Not at all | Slightly | Moderately | Considerably | Extremely |
| 1 | How much do certain types of food/drink cause you discomfort (spicy food, acidic food)? |  |  |  |  |  |
| 2 | How much does your oral condition cause you to limit the types of food/drinks you consume? |  |  |  |  |  |
| 3 | How much do certain food textures cause you discomfort (rough food,  crusty food)? |  |  |  |  |  |
| 4 | How much does your oral condition cause you to limit the textures of the food you consume? |  |  |  |  |  |
| 5 | How much does the temperatures of certain foods/drinks cause you discomfort? |  |  |  |  |  |
| 6 | How much does your oral condition cause you to limit the temperature of the foods/drinks you consume? |  |  |  |  |  |
| 7 | How much does your oral condition cause you to limit your daily oral hygiene routine due to discomfort (brushing, flossing, mouthwash usage)? |  |  |  |  |  |
| **Investigational Product (IP) and Treatment (Including mouthwashes, gels, creams, ointments, injections, tablets, infusions)** | |  |  |  |  |  |
| 1 | How much do you feel you need IP to help you with activities of daily life (talking, eating, etc)? |  |  |  |  |  |
| 2 | How satisfied are you with the IP being used to treat your oral condition? |  |  |  |  |  |
| 3 | How concerned are you about the possible side effects of the IP used to treat your oral condition? |  |  |  |  |  |
| 4 | How much does the use of the IP limit you in your everyday life (routine/the way you apply or take your medications)? |  |  |  |  |  |
| **Social and Emotional** | |  |  |  |  |  |
| 1 | How much does your oral condition get you down? |  |  |  |  |  |
| 2 | How much does your oral condition cause you anxiety? |  |  |  |  |  |
| 3 | How much does your oral condition cause you stress? |  |  |  |  |  |
| 4 | How much does the unpredictability of your oral condition bother you? |  |  |  |  |  |
| 5 | How much does your oral condition cause you to worry about the future (spread of the condition)? |  |  |  |  |  |
| 6 | How much does your oral condition make you pessimistic about the future? |  |  |  |  |  |
| 7 | How much does your oral condition disrupt social activities in your life (social gatherings, eating out, parties)? |  |  |  |  |  |
